# ImmunoNodes – graphical development of complex immunoinformatics workflows

**DOI:** 10.1186/s12859-017-1667-z

**Published:** 2017-05-08

**Authors:** Benjamin Schubert, Luis de la Garza, Christopher Mohr, Mathias Walzer, Oliver Kohlbacher

**Affiliations:** 1 Center for Bioinformatics, University of Tübingen, Tübingen, 72076 Germany; 2 Applied Bioinformatics, Dept. of Computer Science, Tübingen, 72076 Germany; 3 Department of Cell Biology, Harvard Medical School, Harvard University, Boston, MA 02115 USA; 4 Quantitative Biology Center (QBiC), Tübingen, 72076 Germany; 5 Faculty of Medicine, University of Tübingen, Tübingen, 72076 Germany; 6 Biomolecular Interactions, Max Planck Institute for Developmental Biology, Tübingen, 72076 Germany

**Keywords:** Immunoinformatics, KNIME, Workflow

## Abstract

**Background:**

Immunoinformatics has become a crucial part in biomedical research. Yet many immunoinformatics tools have command line interfaces only and can be difficult to install. Web-based immunoinformatics tools, on the other hand, are difficult to integrate with other tools, which is typically required for the complex analysis and prediction pipelines required for advanced applications.

**Result:**

We present ImmunoNodes, an immunoinformatics toolbox that is fully integrated into the visual workflow environment KNIME. By dragging and dropping tools and connecting them to indicate the data flow through the pipeline, it is possible to construct very complex workflows without the need for coding.

**Conclusion:**

ImmunoNodes allows users to build complex workflows with an easy to use and intuitive interface with a few clicks on any desktop computer.

## Background

Immunoinformatics methods have become a vital part of biomedical research. Their applications span a wide variety ranging from basic immunological to translational research, especially in the field of cancer research [1–3]. These applications often involve several methods, varying from pre- and post-processing routines, to complex statistical analysis procedures, and require a high amount of development time. Additionally, the lack of standardized interfaces and data formats renders the use of different tools in the same pipeline difficult. To overcome these problems, several groups have developed web-based workbenches that allow interacting with several different approaches via a unified interface [4, 5]. However, factors such as data volume, speed, robustness, or legal restrictions (e.g., data privacy or restrictions on data sharing), often prevent the use of web-based solutions.

Due to the variety and number of tasks that a typical immunoinformatics analysis conveys, we have developed ImmunoNodes, a set of components, each carrying out one specific task in immunoinformatics (e.g., human leukocyte antigen (HLA) ligand binding prediction or statistical analyses). By chaining several of these tools together one can form a complete data analysis workflow. Workflows not only enable complex automation tasks, but they also increase reproducibility of scientific studies by documenting the complete data analysis in a standardized form.

In this work, we present an immunoinformatics toolbox whose components can be used without transferring data to a central server across the Internet (thus circumventing data privacy restrictions). It enables the user to build complex workflows and offers unified interfaces and data formats. In order to facilitate collaboration between its several components, we have fully integrated ImmunoNodes into the Konstanz Information Miner Analytics Platform (KNIME) [6, 7], an application for visual workflow development. We thus benefit from KNIME’s rich functionality covering data mining, statistics, visualization, chemo- and bioinformatics [8–10], as well as computational proteomics [11–13]. ImmunoNodes provides a wide range of well-known tools for HLA binding prediction, HLA class I antigen processing prediction, HLA genotyping, as well as epitope-based vaccine design including epitope-selection and string-of-beads assembly.

Having integrated ImmunoNodes into such a versatile workflow development environment that KNIME is, we hope to ease its use and thus to spread the application of advanced immunoinformatics tools to a wide range of users.

ImmunoNodes is available for all major platforms (Windows, OSX, Linux) and released under a 3-clause BSD license. It can be directly installed from the KNIME-Community repository and its source code can be found at GitHub (https://github.com/FRED-2/ImmunoNodes). The accompanying Docker image can be found at Docker Hub (https://hub.docker.com/r/aperim/immunonodes).

## Implementation

### KNIME Integration

KNIME is a free, stand-alone, open-source, workflow development framework for personal computers. Out of the box, it includes hundreds of sample workflows, more than 1,000 different tools (nodes) including a wide range of solutions for statistics analysis, data acquisition and visualization [14]. KNIME runs on all major operating systems and can be easily extended by writing plug-ins and extensions. It is thus a popular and widespread platform for data analysis.

The ImmunoNodes framework has dependencies on command line tools that, with some considerable effort, could be imported as KNIME nodes. However, the Generic Knime Node (GKN) extension was developed to assist users to add arbitrary command line tools into KNIME. Instead of asking the end user to focus on writing code to enable the interaction between external command line tools and KNIME, GKN enables pipeline designers to mainly concentrate on describing the tools to be added. This description has to be contained in a Common Tool Descriptor (CTD) file [15]. A CTD file is an XML document defining input data, output data, and all parameters required by each tool. Input and output data types are identified by their MIME content types (e.g., text/xml, application/zip) and parameters can be as simple as a single integer number restricted to a range or as complex as a list of nested values. CTDs also contain a section to map named parameters to command line parameters and thus enable the execution of arbitrary command line tools. We use CTD as an abstraction layer for the description of all tools in ImmunoNodes. The software package Generic KNIME Nodes (GKN) (https://github.com/genericworkflownodes) is then used to automatically generate the KNIME plugins from these abstract representations. Several of the software components used in ImmunoNodes are often difficult to install or are available exclusively for Linux. To address these issues, we have extended GKN to be natively able to execute command line tools provided within a Docker container. Docker is a software project that enables a lightweight virtualization of software applications, which internally allows an easy deployment of fully configured software suites to the end user. Docker also permits the execution of Linux-only third-party immunoinformatics tools on Windows and Mac OS X operating systems and thus gives ImmunoNodes full portability. GKN automatically generates the required Docker calls and handles the interaction between the host system and the virtualized Docker container. The majority of nodes in ImmunoNodes are command line tools written with FRED 2 [16]. FRED 2 is an immunoinformatics Python module that provides standardized interfaces to the immunoinformatics software.

### Node Implementation

ImmunoNodes offers twelve different nodes covering epitope, proteasomal cleavage, and transporter associated with antigen processing (TAP) prediction, distance-to-self calculations of peptides, as well as HLA genotyping (Table 1). It also offers nodes for vaccine design including epitope selection and assembly. Each node wraps a variety of state-of-the art tools, many of which were covered in a recent review on immunoinformatics [17].

**Table 1:** Supported immunoinformatics methods sorted by field of application

Method	Version	Purpose	Reference
Epitope Prediction:			
• BIMAS	1.0	MHC-I binding	[35]
• SVMHC	1.0	MHC-I binding	[36]
• ARB	1.0	MHC-I binding	[37]
• SMM	1.0	MHC-I binding	[38]
• SMMPMBEC	1.0	MHC-I binding	[39]
• Comblib 2008	1.0	MHC-I binding	[40]
• PickPocket	1.1	MHC-I binding	[34]
• NetMHC	4.0	MHC-I binding	[32]
• NetMHCpan	3.0	MHC-I binding	[41]
• HAMMER	1.0	MHC-II binding	[42]
• TEPITOPEpan	1.0	MHC-II binding	[43]
• NetMHCII	2.2	MHC-II binding	[44]
• NetMHCIIpan	3.1	MHC-II binding	[45]
• SYFPEITHI	1.0	T-cell epitope	[46]
• UniTope	1.0	T-cell epitope	[25]
• NetCTLpan	1.1	T-cell epitope	[47]
• Callis propensity	1.0	T-cell epitope/Immunogenicity	[48]
Cleavage Prediction:			
• ProteaSMM (C/S20)	1.0	Cleavage site	[49]
• PCM	1.0	Cleavage site	[50]
• NetChop	3.1	Cleavage site	[51]
TAP Prediction:			
• SVMTAP	1.0	TAP affinity	[50]
• SMMTAP	1.0	TAP affinity	[52]
• Additive matrix	1.0	TAP affinity	[53]
Epitope Selection:			
• OptiTope	1.0	Epitope selection for vaccine design	[21]
Epitope Assembly:			
• TSP approach	1.0	String-of-beads design	[22]
• Spacer design + TSP	1.0	Spacer design	[24]
HLA Typing:			
• OptiType	1.0	MHC-I typing	[54]
• Seq2HLA	2.2	MHC-I/II typing	[55]

### Epitope prediction node

Consumes two files, namely, a text file containing HLA alleles, one per line, in new nomenclature (see http://hla.alleles.org), and a text file either containing protein sequences in FASTA format or short peptide sequences, one per line. Besides specifying the desired epitope length, the user can choose an epitope prediction method from a variety of options (Table 1 - Epitope Prediction). The node returns a tab-separated file containing the predicted score for each peptide and allele.

### Neoepitope prediction node

Consumes a VCF file containing the identified somatic genomic variants, besides a text file containing HLA alleles, and generates all possible neo-epitopes based on the annotated variants contained in the VCF file by extracting the annotated transcript sequences from Ensemble [18] and integrating the variants. Optionally, it consumes a text file, containing gene IDs of the reference system used for annotation, which are used as filter during the neoepitope generation. The user can specify whether frameshift mutations, deletions, and insertions should be considered in addition to single nucleotide variations (default). NeoEpitopePrediction currently supports ANNOVAR [19] and Variant Effect Predictor [20] annotations for GRCh37 and GRCh38 only.

### Cleavage prediction node

Takes a FASTA file and predicts the cleavage probability for each site (Table 1 – Cleavage Prediction). In addition, the user can specify a peptide length, which in turn will alter the output to a tab-separated text file containing peptide sequences of the specified length with their C-terminal cleavage score.

### TAP prediction node

Consumes either a FASTA file or a file containing peptide sequences. Besides the TAP prediction model to use (Table 1 - TAP Prediction), the user can specify the required peptide length (if the input was a FASTA file). Its output is again a tab-separated file containing the peptide sequences and the predicted TAP score.

### HLA typing node

Takes a paired-end or single-end whole exome, whole genome sequence, or RNA-Seq FASTQ files and infers the most likely HLA class I and II genotype depending on the method used (see Table 1 - HLA Typing). The resulting file contains the most likely genotype with one HLA allele per line.

### Epitope selection node

Selects an optimal set of epitopes from a set of candidate epitopes that maximizes the overall predicted immunogenicity. The tool implements OptiTope, an integer linear programming-based epitope selection framework proposed by Toussaint et al. [21]. As input it takes a file containing the results of (Neo)EpitopePrediction and a tab-separated HLA allele file with assigned population frequencies, similar to the type of files that AlleleFrequency can generate. Optionally, EpitopeSelection accepts a tab-separated file containing the epitope sequences of the EpitopePrediction result with assigned conservation scores. The user can specify the number of epitopes to select, the percentage of HLA alleles and antigens that have to be covered by the selected epitopes, and a HLA binding threshold that specifies at what point a peptide is considered to bind to a specific HLA allele. If an epitope conservation file is provided, the user can define a minimum conservation to filter the epitopes with.

### Epitope assembly node

Assembles a set of epitopes into an optimal string-of-beads polypeptide vaccine construct. It consumes a peptide list and generates a traveling salesman problem (TSP) instance as described in [22]. Each node of the underlying fully connected graph represents a peptide, each edge’s weight expresses the cleavage probability of the connected epitopes predicted by the user specified cleavage site prediction model. Solving the TSP instance yields a string-of-beads construct that has the highest probability to be fully recovered. The user can either specify to solve the TSP instance either optimally via integer linear programming by using the CBC solver (https://projects.coin-or.org/Cbc), or to obtain an approximate solution by using the Lin-Kernighan heuristic [23]. Optionally, the user can specify a weight parameter (which defaults to 0) that activates and weights an additional term of the objective function. The additional term represents the non-junctional cleavage likelihood, which, by providing a weight greater to zero, will be minimized, whilst the junction cleavage likelihood will be maximized.

### Spacer design node

Generates a string-of-beads design similar to the EpitopeAssembly node but also constructs optimal spacer sequences maximizing the cleavage probability of the desired epitopes. The tool consumes a peptide list and generates a TSP instance. Additionally, it calculates short spacer sequences connecting two epitopes to increase the cleavage likelihood of the epitopes while simultaneously reducing the formation of neoepitopes [24]. The user has to specify an epitope prediction model in addition to the required cleavage site model. The output, like in EpitopeAssembly, is a FASTA file containing the designed string-of-beads vaccine.

### Distance-to-self nodes

Can be used to calculate the distance of a given $l$ -mer peptide to the whole human proteome or a user-defined set of proteins. To this end, distance-to-self uses a memory efficient trie-based data structure to hold the reference proteome or any set of protein sequences and to query it with a target peptide as previously described in [25]. The distance calculation is based on a distance measure derived from a transformed BLOSUM substitution matrix and lies between 0 (most similar) and 1 (most dissimilar). ImmunoNodes provides two distance-to-self nodes: Distance2SelfGeneration and Distance2SelfCalculation. Distance2SelfGeneration can be used to generate custom reference tries for a given protein FASTA and the desired length of peptides in the trie, while Distance2SelfCalculation calculates the distances of the $k$ closest reference peptides of a custom build, or pre-calculated reference trie for a list of peptides given in a tab-separated file. There are four pre-calculated reference tries generated from all 8−, 9−, 10−, and 11−mers of the human reference proteome (Uniprot, TrEMBL, accesse 04/07/2016).

### Allele frequency node

Is a very simple node that takes a list of HLA alleles and assigns the probability that a given HLA allele occurs in the user-specified geographic region or population extracted from dbMHC [26]. The output is a tab-separated file, each row containing an HLA allele and its probability of occurrence in the given region or population.

### Epitope conservation node

Consumes a multiple sequence alignment, calculates the consensus sequence and generates peptides of a user specified length. In addition to that, the multiple sequence alignment is used to calculate peptide conservation, which is defined as the product of column-wise conservation of the MSA. In the case of multiple epitope origins the maximum epitope conservation is reported [21]. The output is a tab-separated file containing the peptide sequences and their conservation.

## Results

### Example workflow 1: HLA ligandomics analysis pipeline

**Fig. 1:**
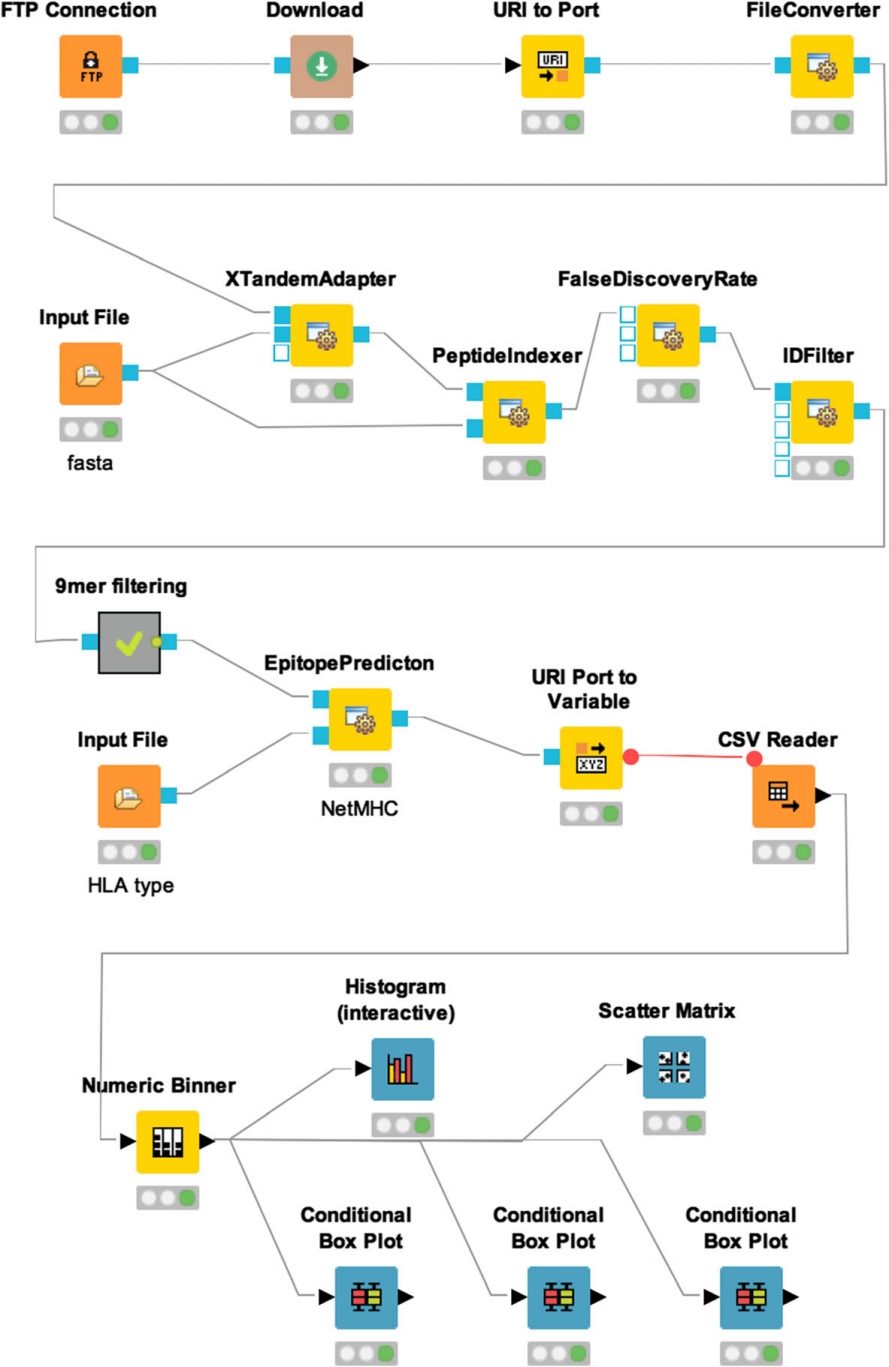
HLA ligandomics workflow combining native KNIME, OpenMS, and ImmunoNodes nodes. The workflow extracts MS data from PRIDE (FTP Connection and Download node) and performs mass spectra identification with the peptide search engine X!Tandem (XTandemAdapter), annotates the results with details of the given target/decoy database (PeptideIndexer), calculates false discovery rates (FalseDiscoveryRate) and filters for 5% FDR (IDFilter) using OpenMS’ nodes. The identified peptides are annotated with their respective binding affinity predicted by NetMHC using the EpitopePrediction node. Finally, simple summary statistics and visualizations are generated with the use of native KNIME nodes

Recently, high throughput methodologies based on liquid chromatography and mass spectrometry (MS) have been successfully used to identify therapeutic targets for cancer immunotherapies [27–29]. Here, we present a peptide identification workflow for ligandomics analysis using OpenMS [30] and ImmunoNodes (Fig. 1, http://www.myexperiment.org/workflows/4947). At the same time, this workflow will exemplify the synergistic effects of combining native KNIME nodes, other community extensions, and ImmunoNodes.

First, ligandomics data of JY cell lines are downloaded from PRIDE [31] via an FTP download node. Then, peptide identification at 5% FDR is applied using OpenMS nodes [11]. The resulting peptides are then annotated with their predicted binding affinity using ImmunoNodes’ EpitopePrediction with NetMHC [32] and simple statistics of the predicted binding affinities are calculated and visualized using native KNIME nodes.

### Example workflow 2: population-based vaccine design against Zika virus

**Fig. 2:**
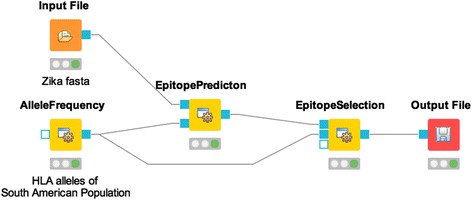
Population-based vaccine design workflow in KNIME. AlleleFrequency is used to specify the geographical region or population of interest and returns a tab-separated list of HLA alleles with their corresponding occurrence probability within the selected population. This file, together with a FASTA file containing protein sequences, or a file containing peptides is used as input to EpitopePrediction, which generates a file containing the predicted binding affinities of the (generated) peptides and the selected HLA alleles. This file, in turn, is used as input to EpitopeSelection, which selects a user-defined number of epitopes out of the candidate pool and writes these together with other statistics into an output file

To demonstrate the usage of ImmunoNodes for vaccine design, we extracted all 221 partially and 30 fully sequenced genomes of Zika virus from the Virus Pathogen Resource database [33] (access 02/22/2016). Epitope prediction was performed with PickPocket [34] using HLA alleles with a minimal prevalence of 1% in the South American population and nine-mer peptides generated from the extracted protein sequences. The candidate epitopes were filtered based on a binding threshold of 500 nM, and EpitopeSelection was allowed to select up to ten epitopes that guaranteed the maximal obtainable antigen and HLA allele coverage (Fig. 2, http://www.myexperiment.org/workflows/4948).

The ten selected epitopes (Table 2) covered more than 95% (20 of 21) of the HLA alleles prevalent in the South American population, as well as 92% (287 of 312) of the extracted Zika antigens. The alleles of HLA-A, −B, −C of the South American population could be covered by 100%, 83%, and 100% respectively with the selected epitopes, resulting in a 99% population coverage (i.e., the probability that a person of the South American population carries at least one HLA allele that is covered by the vaccine is 99%).

**Table 2:** Selected Zika epitopes for potential vaccine design using EpitopeSelection

Sequence	Fraction of Objective	Allele Coverage
FHTSVWLKV	0.09	B*39:01 B*39:06
FTNLVVQLI	0.12	A*02:01
TMSYECPML	0.12	A*02:17 A*02:01 A*02:04
MAMATQAGV	0.14	C*03:04 C*03:03 C*15:03 C*15:02 A*02:01
YRVMTRRLL	0.10	C*07:02
VMAQDKPTV	0.12	A*02:17 A*02:01 A*02:04
GPIRMVLAI	0.07	B*35:06 B*35:04 B*51:01 B*35:01 B*51:04
AWLMWLSEI	0.08	A*24:02 A*24:03
QEGAVHTAL	0.07	B*40:04 B*40:02
FALAWLAIR	0.07	A*68:03

‘Fraction of Objective’ illustrates the proportional contribution of each epitope to OptiTope’s objective function

## Conclusion

The complexity and development time of accurate, state-of-the-art immunoinformatics tasks is high. To maximize quality in the results and to decrease implementation time, it is common that immunoinformatics software makes use of already existing, thoroughly tested libraries. Unfortunately, the installation and configuration of the different components of such pipelines tends to be non-trivial and often exceeds the technical capabilities of many end users.

Having these aspects in mind, we developed ImmunoNodes, an immunoinformatics framework that covers essential tasks of pipelines such as epitope discovery, HLA inference, antigen processing, and vaccine design. Structuring complex scientific tasks into a collection of small, easily executable, simpler computations (i.e., a pipeline or workflow) brings the benefit of adding a certain degree of reproducibility, an aspect desired in all scientific endeavors. Being fully integrated into KNIME using GKN, it enables a wide audience to develop complex analysis workflows without the need of having mastered a programming language. Also, the complexity of installation and configuration of required third-party libraries has been lifted from the end user as a result of the provided Docker images. We therefore are confident that ImmunoNodes will enable a wide range of users to develop innovative and complex pipelines, thus spreading the usage of state-of-the-art immunoinformatics approaches.

## Abbreviations

CTD: Common tool descriptor
GKN: Generic KNIME node
HLA: Human leukocyte antigen
IDE: Integrated development environment
IEDB: Immune epitope database
KNIME: Konstanz information miner
TAP: Transporter associated with antigen processing
TSP: Traveling salesman problem
VCF: Variant calling format
XML: Extensible markup language
